# Towards a new level of quantitative treatment of 3D electron diffraction data – in-pattern optical distortions

**DOI:** 10.1107/S2052252522010326

**Published:** 2022-11-01

**Authors:** Tatiana E. Gorelik

**Affiliations:** a Structure and Function of Proteins, Helmholtz Centre for Infection Research, Inhoffenstraße 7, Braunschweig, Germany; b Helmholtz Centre for Infection Research, Helmholtz Institute for Pharmaceutical Research Saarland, Saarland University Campus, Saarbrucken, 66123, Germany

**Keywords:** 3D electron diffraction, microED, distortions, lattice parameters

## Abstract

Electron diffraction patterns unavoidably contain in-plane distortions introduced by electromagnetic lens systems. Geometric correction of different distortion types allows the accuracy of lattice parameter determination to be improved in 3D electron diffraction data.

The availability of aberration correctors for transmission electron microscopes leads to a significant improvement of the achievable resolution (Batson *et al.*, 2002 ▸); for beam-stable materials, sub-ångstrom resolved images can routinely be obtained using modern TEMs. Lens aberrations are inherent properties of electromagnetic elements (Haider *et al.*, 1998 ▸). Consequently, their effect is also present in electron diffraction patterns, which are formed at the back focal plane of the objective lens. In addition to the properties of the objective lens, electron diffraction patterns are affected by the characteristics of the diffraction/projector lens system and by the deviation of the electron beam from the optical axis of the microscope, whether intended (precession electron diffraction) or unintended (owing to misalignment).

Electron crystallography based on the analysis of 3D electron diffraction data is a rapidly developing field, demonstrating remarkable achievements in structure analysis of nano- and microcrystals of diverse nature – from minerals to proteins (Gemmi *et al.*, 2019 ▸; Gruene & Mugnaioli, 2021 ▸). Different instrumental approaches including dedicated electron diffractometers (Heidler *et al.*, 2019 ▸) are available. The data reduction is performed either using dedicated programs for electron diffraction (Kolb *et al.*, 2011 ▸; Gemmi & Oleynikov, 2013 ▸; Palatinus *et al.*, 2019 ▸; Wan *et al.*, 2013 ▸) or X-ray software [*e.g.* *XDS* (Kabsch, 2010 ▸), *DIALS* (Winter *et al.*, 2022 ▸), *MOSFLM* (Battye *et al.*, 2011 ▸)]. The structure solution is typically performed with X-ray programs [*SHELXT* (Sheldrick, 2015 ▸), *SIR* (Burla *et al.*, 2015 ▸), *Superflip* (Palatinus & Chapuis, 2007 ▸), *Phaser* (McCoy *et al.*, 2007 ▸)]. The success of dynamical refinement, reliably delivering hydrogen-atom positions (Palatinus *et al.*, 2017 ▸) and the absolute configuration (Brázda *et al.*, 2019 ▸), clearly demonstrated the potential and necessity of *dedicated approaches* for the quantitative treatment of effects specific to electron scattering.

Aberrations of electron optical systems bring a distortion field in 2D electron diffraction patterns. These distortions unavoidably propagate into the 3D ED data and deform the complete reciprocal space. The presence of the distortions usually does not hamper the indexing, but may introduce inaccuracy of the lattice parameters, and can lead to improper integration of intensities due to displacement of reflection predictions when mapped back onto 2D frames.

In this issue of **IUCrJ** , Brázda *et al.* (2022 ▸) present a comprehensive analysis of different types of distortions present in electron diffraction patterns, their propagation into the reconstructed reciprocal space, and elaborated methods for their detection and correction. These methods are already implemented in the new version of *PETS2* – widely used dedicated software for 3D ED data reduction (Palatinus *et al.*, 2019 ▸).

In a general form, the positional shift of a point due to a distortion can be separated into its radial 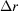 and tangential 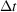 components [Brázda *et al.*, 2022 ▸; equations (6) and (7)]. The axial symmetry of the optical system sets selection rules on the orders of the circular harmonic and polynomial terms, so that *n* + *m* should be odd (Smith, 2007 ▸; Linck, 2022 ▸). For different distortion types, the formulae for radial and tangential distortion components can then be deduced (Table 1 ▸), containing different power dependence on *r*. Magnification, rotation and elliptical distortions [Fig. 1 ▸(*b*)] contain linear dependence on *r*, parabolic [Fig. 1 ▸(*c*)] is quadratic with *r*, and barrel [Fig. 1 ▸(*d*)], pincushion [Fig. 1 ▸(*e*)] and spiral [Fig. 1 ▸(*f*)] have cubic dependence on *r*. The angular part of the distortions, which depends on the azimuth φ, is zero-order for magnification, rotation, spiral and barrel–pincushion distortions (they do not depend on φ), first-order for parabolic distortion (the period is 2π), and second-order for elliptical distortion (the period is π).

An important point of the study of Brázda *et al.* (2022 ▸) is the elaboration of the relationship between different types of distortions and the possibility to correct for them for a given dataset without any additional information. The *magnification distortion* is equivalent to the scaling factor and is fully correlated with the lengths of the unit-cell vectors. This makes it impossible to decouple from the lengths of the unit-cell vectors, and correct for, without any additional knowledge. The *rotation distortion* correlates completely with the tilt axis position in the frames, which also makes it difficult to separate, yet it does not affect the values of the unit-cell vectors, solely resulting in the rotation of the orientation matrix.

The *elliptical distortion* represents a complex case: the component along the tilt axis (and orthogonal to it) is equivalent to stretching or contracting of the complete reciprocal space along the tilt axis, and therefore distorts the lattice perfectly linearly, resulting in the change of the unit-cell parameters (*i.e.* it is fully correlated with the unit-cell metric). The component away from the tilt axis does lead to the distortion (twisting) of the lattice, which can be corrected for. For a full correction of elliptical distortion, additional knowledge on the crystal class or an additional dataset from a differently oriented crystal is necessary (under the assumption that the elliptical distortion is the same for the two datasets). Distortions with quadratic and cubic dependence on *r* described by Brázda *et al.* (2022 ▸) lead to specific deformations of the reciprocal lattice, and can be refined simultaneously with the unit-cell parameters.

Accounting for the specified distortions in electron diffraction data has a number of implications. Apart from the obviously more accurate unit-cell parameters, enabling the crystal system determination, and improving the integration of intensities, the final structure models provide a more accurate bond geometry, and finally, better accuracy of the unit-cell parameters improving the findability of the structures within the databases.

In the first publication of the series, Brázda *et al.* (2022 ▸) consider the effect of distortion within 2D frames. The next step is the correction for misalignment of individual frames. It has been demonstrated that the in-frame distortion correction combined with the frame orientation allowed for better visibility of hydrogens in cobalt aluminophosphate (Brázda & Palatinus, 2022 ▸) compared with uncorrected data extraction reported in 2017 (Palatinus *et al.*, 2017 ▸), and significantly improved (Krysiak, 2022 ▸) data reduction and structure analysis of hydrous layer silicate RUB-6 (Krysiak *et al.*, 2020 ▸). We therefore look forward to seeing a significant step forward in the accuracy of electron diffraction structure analysis in general, driven by the implementation of more accurate models of electron diffraction data geometry.

## Figures and Tables

**Figure 1:**
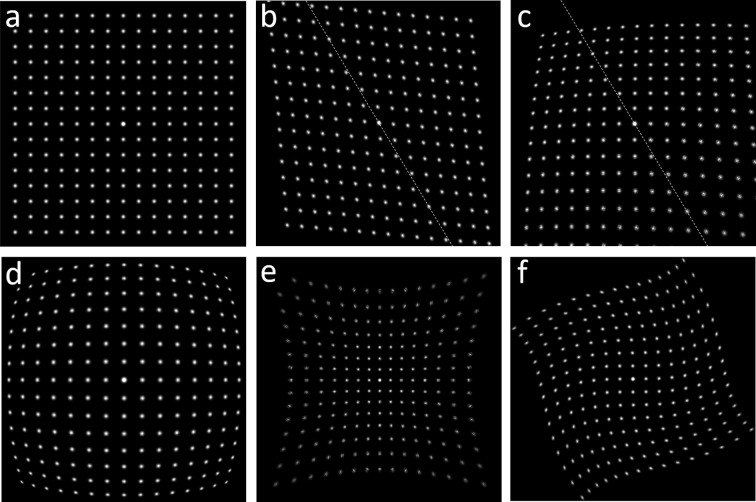
Different types of distortion in electron diffraction patterns: (*a*) original undistorted image, (*b*) elliptical distortion with the distortion axis inclined 30° away from the vertical axis, (*c*) parabolic (coma) distortion, (*d*) barrel with a negative 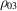 parameter, (*e*) pincushion with a positive 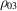 , (*f*) spiral distortion.

**Table 1:** Different types of distortions observed in 2D electron diffraction patterns – formulae for the radial and tangential displacements 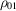
 , 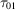 , 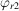 , 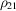 , 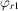 , 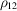 , 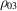 , 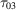 are scalar coefficients.

Distortion types	Radial and tangential distortion components
Magnification/scaling factor	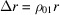 , 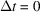
In-plane pattern rotation	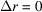 , 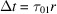
Elliptical	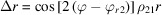 , 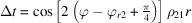
Parabolic	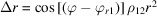 , 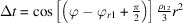
Barrel–pincushion	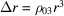 , 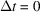
Spiral	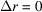 , 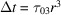
